# Surgical Approach to a Rare Forearm Morel-Lavallée Lesion: Diagnostic and Management Challenges in the Upper Extremity

**DOI:** 10.7759/cureus.105329

**Published:** 2026-03-16

**Authors:** Dayanna A Zuluaga, Sohail Khan, Ammr Al-Houssan, Jacob I Jabbour

**Affiliations:** 1 Department of Primary Care, Touro College of Osteopathic Medicine, Middletown, USA; 2 Department of Surgery, University of Connecticut School of Medicine, Farmington, USA; 3 Department of Hand Surgery, University of Connecticut School of Medicine, Farmington, USA; 4 Department of Hand Surgery, Hospital of Central Connecticut, New Britain, USA

**Keywords:** closed degloving injury, dermal allograft, diagnostic challenge, forearm degloving injury, morel-lavallée lesion, post-traumatic hematoma, soft tissue injury, surgical debridement, upper extremity trauma, wound reconstruction

## Abstract

Morel-Lavallée lesions (MLLs) are closed soft-tissue degloving injuries most often reported in the pelvis and lower extremity after high-energy shear trauma. Upper extremity (UE) involvement is exceedingly rare and frequently misdiagnosed as cellulitis or abscess.

We report a 49-year-old Black male with a history of psychiatric disorders and intravenous drug use (IVDU) who presented to the Hospital of Central Connecticut’s Emergency Department (ED) with a swollen, erythematous, and painful right forearm one week after blunt trauma. Contrast-enhanced computed tomography (CT) was inconclusive, but ultrasonography demonstrated a 20-cm unencapsulated suprafascial fluid collection. Magnetic resonance imaging was not obtained, given the emergent presentation requiring urgent intervention. Empiric intravenous vancomycin and piperacillin-tazobactam were initiated, and within 12 hours, spontaneous drainage confirmed infection and prompted urgent incision, drainage, and wide debridement, which established the definitive diagnosis. Intraoperative findings revealed a shearing-induced hematoma with overlying necrotic tissue, and pathology was consistent with an infected MLL. Given the large defect, marginal wound bed viability, and the patient’s refusal of split-thickness skin grafting (STSG) due to concerns about donor-site morbidity, a dermal allograft skin substitute was used to provide immediate coverage without graft harvest. The substitute integrated successfully, and longitudinal follow-up demonstrated progressive epithelialization with durable closure.

This case expands the limited literature on UE MLLs by illustrating how delayed history, atypical anatomy, and equivocal imaging can obscure diagnosis and delay definitive care. Forearm MLLs may closely mimic cellulitis or abscess, underscoring the importance of maintaining diagnostic suspicion for closed degloving injuries in patients presenting with post-traumatic UE swelling.

Once infection and necrosis are evident, timely surgical intervention is essential to achieve source control and preserve soft-tissue viability. Management should be individualized based on lesion characteristics and patient-specific risk factors. In this case, use of a dermal allograft provided durable closure in a high-risk patient who declined STSG, demonstrating a viable reconstructive alternative. Early recognition and tailored operative strategy are critical to reducing morbidity in anatomically uncommon presentations such as the forearm.

## Introduction

Morel-Lavallée lesions (MLLs) are rare closed soft-tissue degloving injuries caused by shearing forces that separate subcutaneous tissue from the underlying fascia, creating a potential space filled with hemolymphatic fluid and necrotic fat. First identified by the French physician Maurice Morel-Lavallée in the mid-19th century, these lesions are most commonly associated with high-energy trauma in areas prone to shear, such as the pelvis and lower extremities, with upper extremity (UE) involvement exceedingly rare [1]. Fluid accumulation in an MLL can become encapsulated within a fibrous pseudocapsule, which prevents spontaneous resorption and predisposes to chronic inflammation, persistent swelling, and secondary infection, if untreated [2,3]. The distinction between acute and chronic MLLs carries direct management implications, as non-encapsulated lesions may respond to conservative treatment, but encapsulated or infected lesions generally demand operative intervention.

A review of 204 cases across 195 patients documented no forearm MLLs [4], and only four cases involving the shoulder and elbow were reported among 87 patients [5]. The rarity and nonspecific presentation of UE MLLs, manifesting as fluctuating swelling, localized pain, and skin discoloration, often lead to misdiagnosis as an abscess and delay appropriate management [6]. Imaging and operative findings are therefore critical in establishing the diagnosis, though clinical suspicion remains paramount, particularly when imaging is equivocal. Magnetic resonance imaging (MRI) remains the modality of choice, providing detailed visualization of lesion contents and extent, especially in chronic cases with pseudocapsule formation [7]. In acute or emergent presentations, practical constraints may preclude MRI, necessitating reliance on ultrasonography and clinical judgment. Ultrasonography offers a rapid, cost-effective assessment in acute or resource-limited settings.

This report describes a rare forearm MLL confirmed by imaging, intraoperative features, and pathology, complicated by infection. By outlining the clinical course, this case contributes to the limited literature on UE MLLs and highlights the diagnostic complexity, therapeutic decision-making, surgical intervention, and outcomes in an unusual anatomic location, guiding surgeons who may encounter similar presentations.

## Case presentation

Presentation

A 49-year-old Black male with a history of psychiatric disorders and intravenous drug use (IVDU) presented with a swollen, erythematous, and painful right forearm to the emergency department (ED) at the Hospital of Central Connecticut in New Britain, CT. The patient delayed disclosing trauma, later reporting that symptoms began one week earlier after sustaining a high-energy compressive injury when an approximately 136-kg individual fell directly onto his forearm. He described progressive worsening despite over-the-counter analgesics. Notably, the severity of pain appeared disproportionate to the visible cutaneous changes, raising early concern for a deeper subcutaneous process beyond simple cellulitis. Intermittent serous drainage from the central forearm lesion was noted, accompanied by tactile fevers and chills during the preceding 24 hours, though he had not recorded a temperature. He denied IVDU within the past month, though the initial history was incomplete due to delayed disclosure of trauma.

Examination

On arrival, he was afebrile, normotensive, and in no acute distress. The volar forearm demonstrated marked edema and fluctuance extending distally to the wrist, with overlying cellulitic changes, central eschar, and scant non-purulent drainage. Distal neurovascular function was preserved with intact sensation and motor strength. Laboratory results revealed leukocytosis (WBC = 18.5 × 103/µL) and elevated lactate (2.4 mmol/L).

Imaging

**Figure 1:**
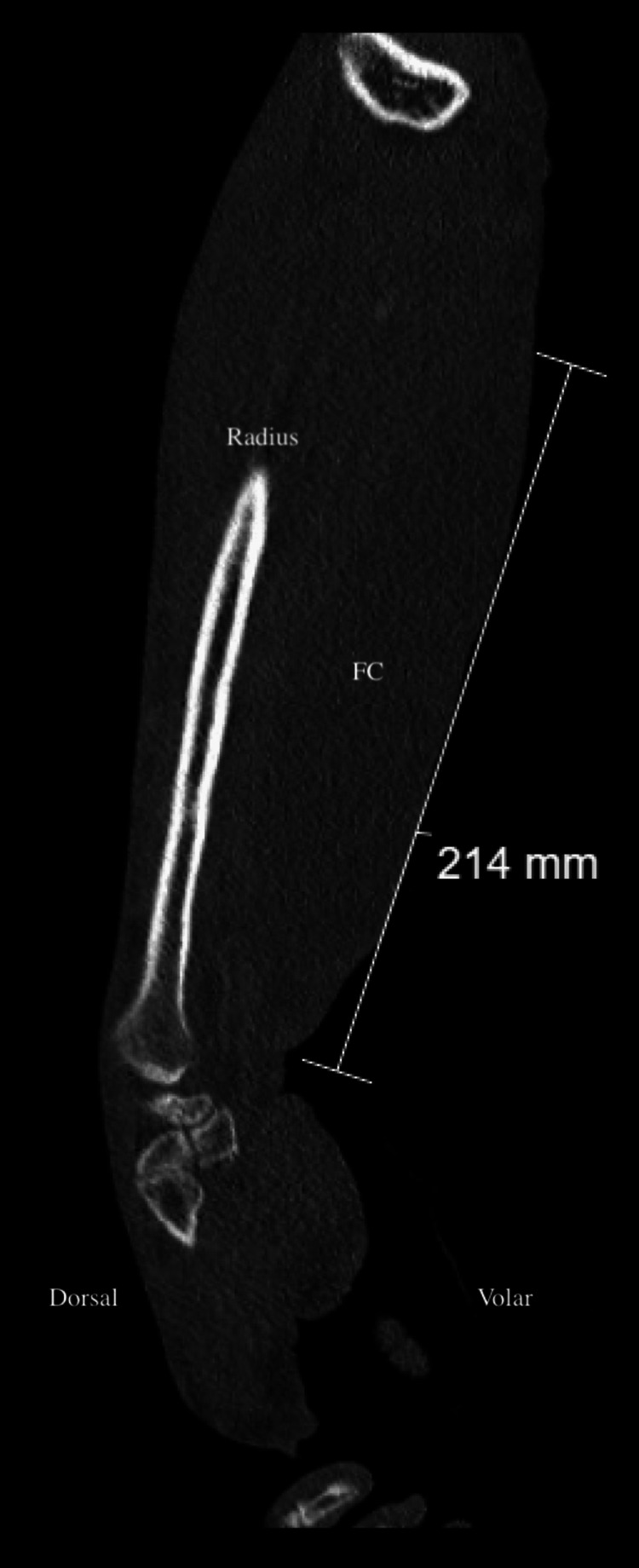
Imaging of the right forearm to evaluate the suspected Morel-Lavallée lesion (MLL). Contrast-enhanced computed tomography scan showing extensive soft tissue edema, though inconclusive due to motion artifacts, which prevented clear visualization of a defined lesion. FC: fluid collection.

**Figure 2:**
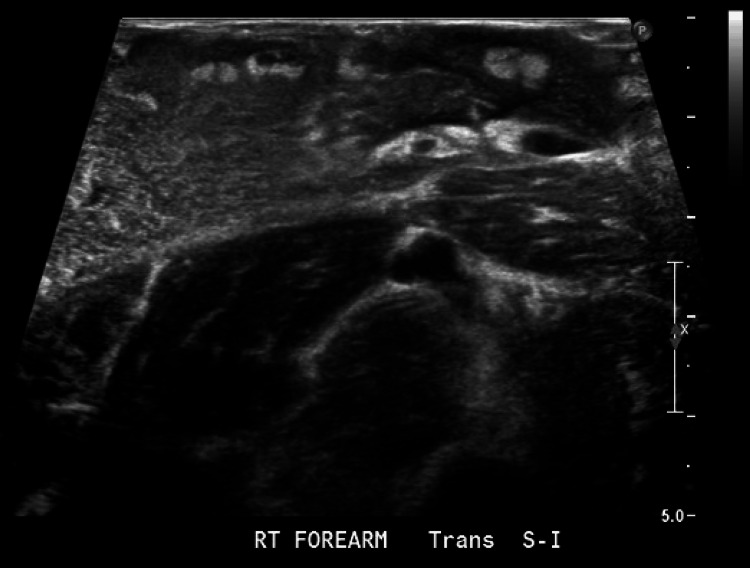
Imaging of the right forearm to evaluate the suspected Morel-Lavallée lesion (MLL). Ultrasonography image revealing a large, 20 cm unencapsulated fluid collection superficial to the forearm musculature, consistent with a hematoma and supporting the diagnosis of MLL.

The initial differential diagnoses included post-traumatic entities like hematoma, abscess, myositis ossificans, early muscular necrosis, cellulitis, and, less likely, soft-tissue sarcoma. Plain radiographs showed no osseous abnormalities or foreign bodies. A contrast-enhanced computed tomography (CT) scan was limited by soft-tissue edema and motion artifact. Subsequent ultrasonography, performed and compared alongside the CT, revealed a 20-cm unencapsulated, suprafascial fluid collection superficial to the musculature and separate from the fascial compartments. No rim enhancement, loculations, or deep pockets were observed sonographically. Although the ultrasonographic appearance was compatible with a hematoma, the lesion’s location, extent, and associated shearing mechanism raised strong clinical suspicion for a UE MLL complicated by infection (Figures 1, 2). MRI, the gold-standard imaging modality for MLL characterization, was deferred given the acute clinical trajectory and the emergent need for source control, which took precedence over further diagnostic refinement.

Management

**Figure 3:**
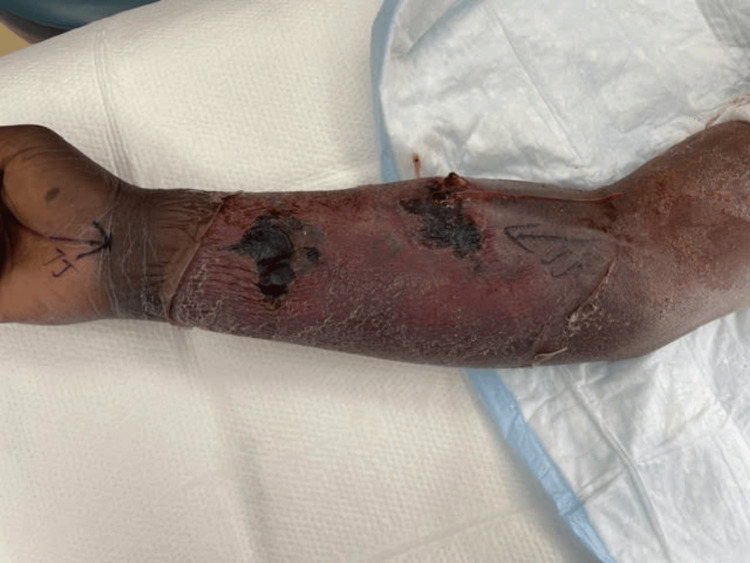
Preoperative clinical photograph of the right forearm. Forearm displays marked edema, fluctuance, and erythema prior to the initial surgical drainage, highlighting the extensive swelling and skin discoloration associated with the lesion.

The large lesion raised concern on presentation, but diagnostic certainty was limited by inconclusive imaging and delayed disclosure of trauma. Empiric intravenous vancomycin and piperacillin-tazobactam were initiated during evaluation. Within 12 hours of admission, spontaneous drainage of approximately 200 mL of sanguinopurulent fluid occurred, confirming infection and delineating the lesion’s extent. This prompted urgent surgical intervention (Figure 3).

**Figure 4:**
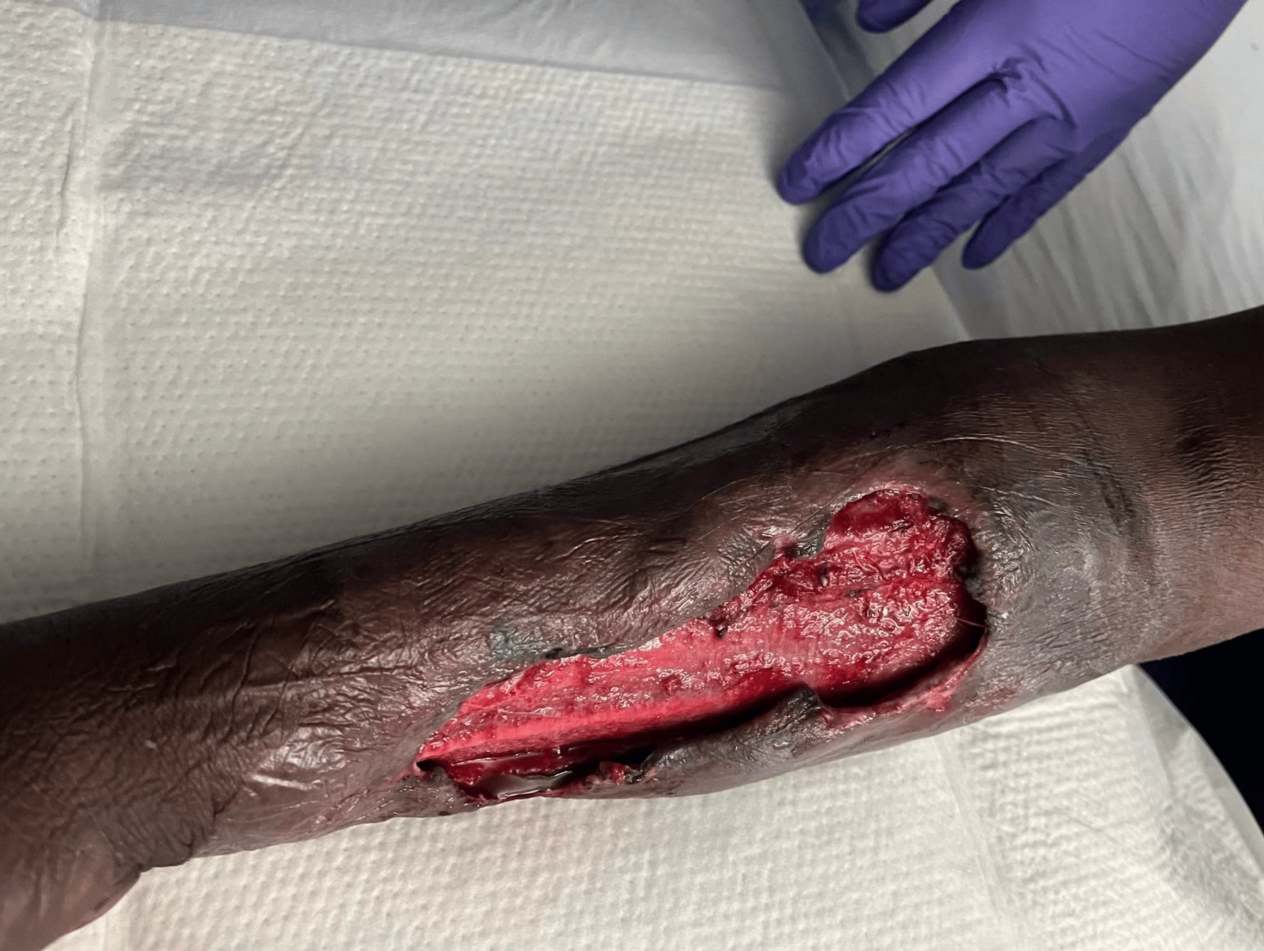
Intraoperative findings during the first surgical intervention. Post-debridement image showing extensive removal of necrotic tissue from the forearm.

**Figure 5:**
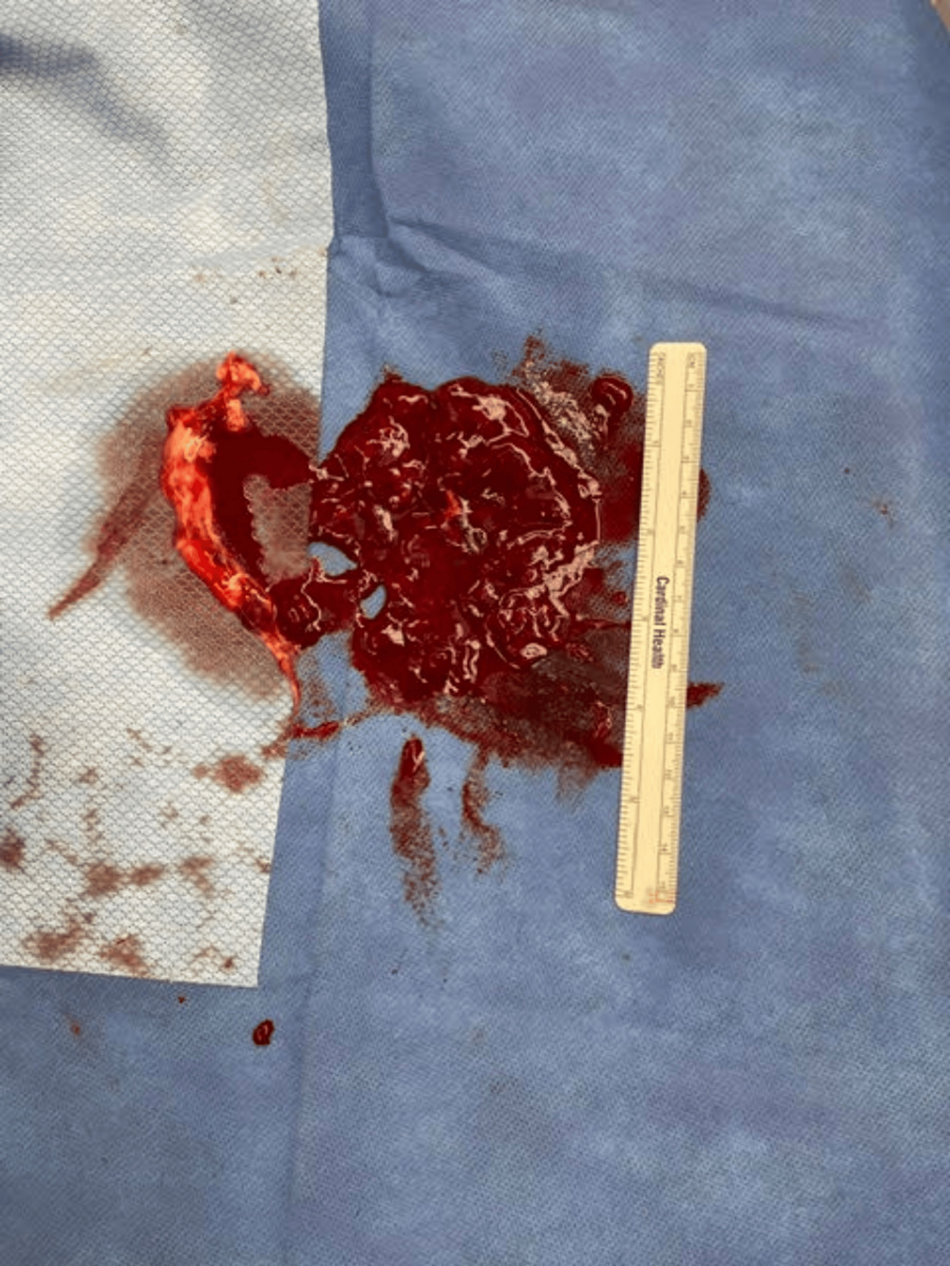
Intraoperative findings during the first surgical intervention. Representative sample of hematoma and necrotic tissue excised during the initial incision and drainage procedure.

The initial operation consisted of incision and drainage, with operative exploration yielding an additional 150 mL of fluid (Figures 4, 5).

**Figure 6:**
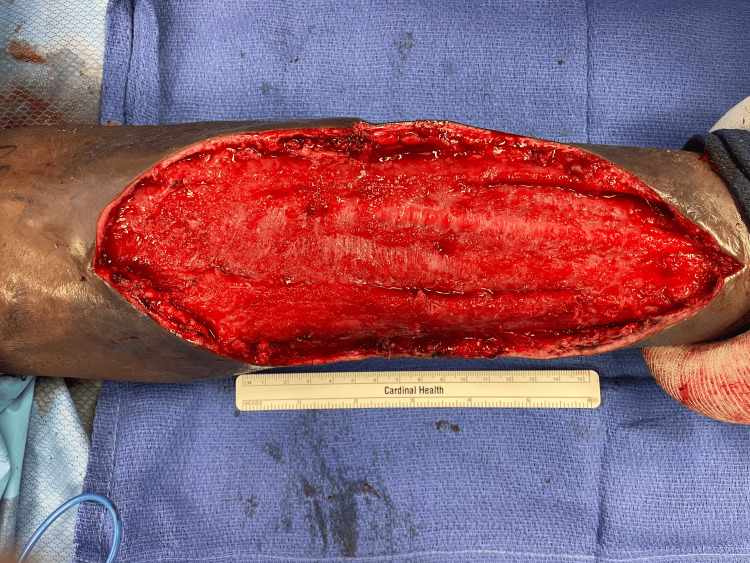
Intraoperative photograph from the second surgery. Definitive debridement of the wound measuring 22 x 10 cm.

**Figure 7:**
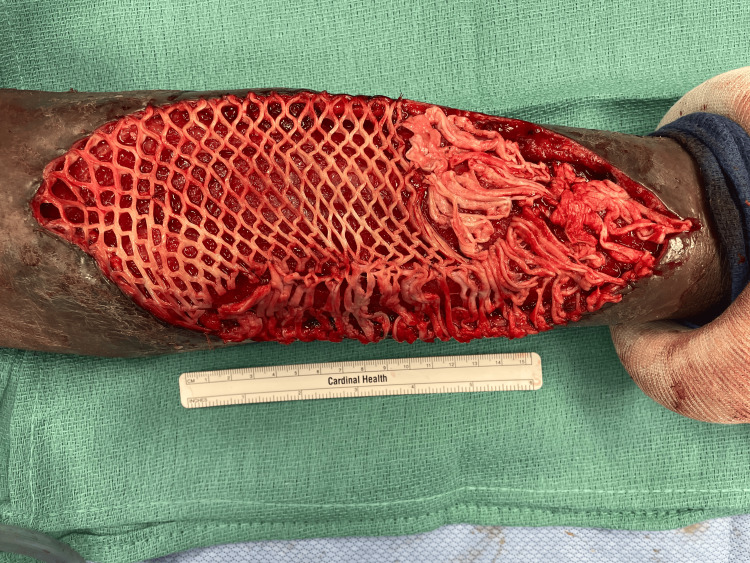
Intraoperative photograph from the second surgery. Wound prepared for the application of a skin substitute graft to facilitate wound healing and coverage of the defect.

A staged, definitive debridement was performed as a second procedure, during which a large suprafascial cavity with overlying devitalized, necrotic skin was identified, necessitating a 22 × 10 cm excisional debridement of skin and subcutaneous tissue (Figures 6, 7).

Pathology was consistent with a UE MLL, demonstrating an infected hematoma with necrotic soft tissue. Together with intraoperative cultures growing group A Streptococcus (*S. pyogenes*), these results reinforced the diagnosis of an infected UE MLL rather than a primary abscess, when integrated with the shearing mechanism, imaging, and surgical assessment. MLLs are well recognized to become colonized or infected, particularly in the perioperative setting. Following debridement and microbiologic confirmation, attention turned to reconstructive management of the significant defect.

Reconstruction and follow-up

Given the large size of the defect, marginal wound bed viability, and ongoing contamination risk, split-thickness skin grafting (STSG) was considered but ultimately declined by the patient, who expressed concern about donor-site morbidity. Additionally, his reluctance toward follow-up care raised further concern for postoperative nonadherence, graft failure, and excluded negative pressure wound therapy as a viable option given the requisite outpatient monitoring and device maintenance. A GraftJacket (Wright Medical Technology, Arlington, TN) acellular dermal allograft skin substitute, measuring 4 × 8 cm with 2-3 mm thickness and meshed with a 3:1 mesher, was therefore selected to provide immediate coverage without graft harvest and to facilitate progressive wound contraction. The graft integrated successfully, resulting in stable coverage.

**Figure 8:**
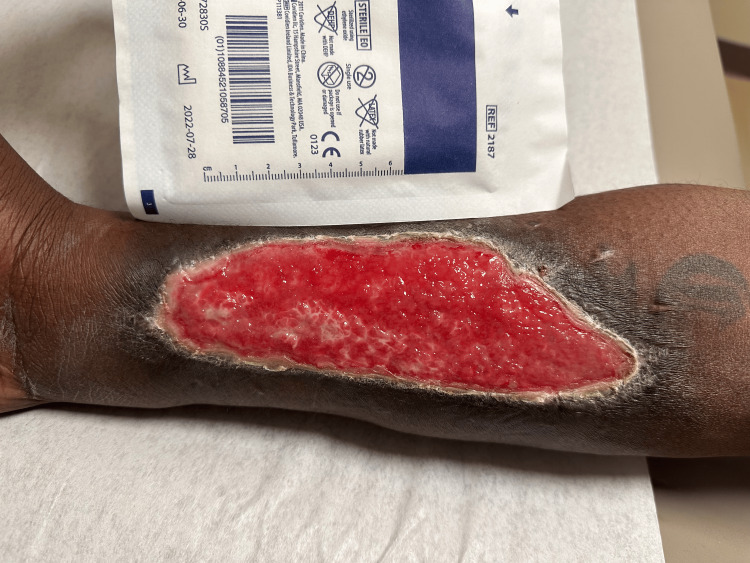
Forearm eight weeks post surgery. The wound demonstrates significant epithelialization with a reduction in size to approximately 15 x 6 cm, showing progressive wound healing from the periphery.

At eight weeks, the wound had contracted to approximately 15 × 6 cm with epithelialization from the periphery (Figure 8).

**Figure 9:**
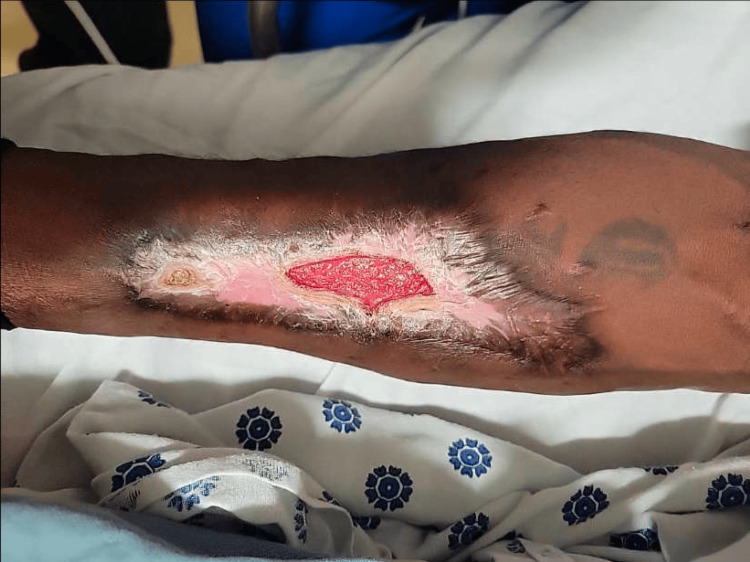
Clinical assessment at five months postoperatively. The forearm shows a healthy wound bed with further epithelialization and a reduction in wound size to approximately 7 x 3 cm, suggesting near-complete healing without open wounds.

**Figure 10:**
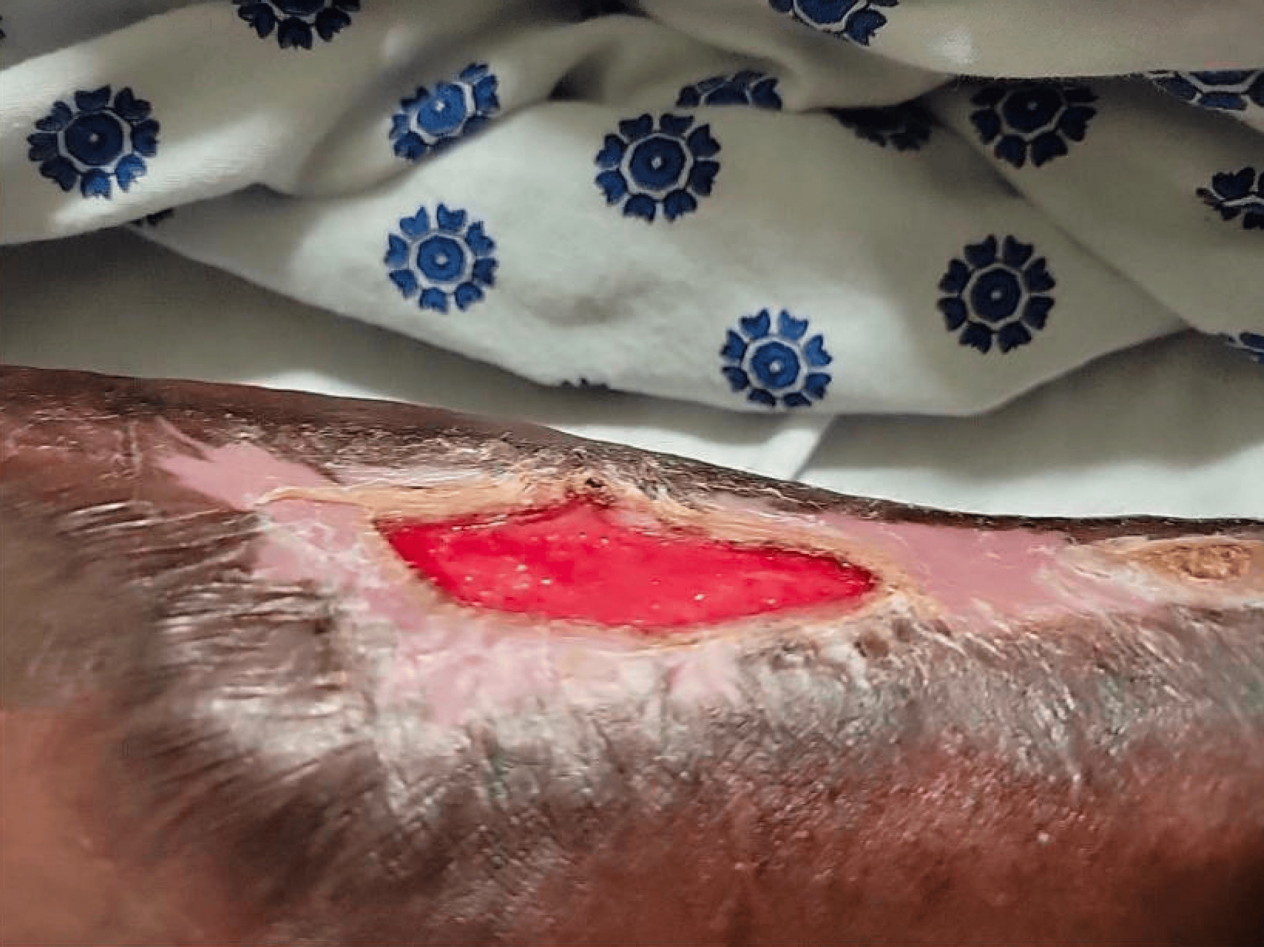
Clinical assessment at five months postoperatively. Close-up of the forearm showing minimal residual wound size indicative of successful integration of previous interventions.

At five months, during an unrelated ED visit, the wound bed was noted to be healthy and measured 7 × 3 cm (Figures 9, 10).

By seven months, serial ED evaluations documented complete epithelialization without residual open wounds. This reconstructive strategy provided durable closure in a patient at high risk for graft failure, underscoring the importance of tailoring wound coverage to defect characteristics and patient-specific risk factors, including inconsistent follow-up.

## Discussion

This case illustrates a rare forearm manifestation of an MLL, an anatomic location that remains poorly described in the literature. Between 2017 and 2025, only four UE cases have been reported, highlighting the exceptional rarity of this presentation [1,2,6,7]. The infrequency of UE MLLs, combined with their atypical clinical features, heightens the risk of delayed recognition and misdiagnosis as cellulitis or abscess. MLLs are closed post-traumatic degloving injuries in the subcutaneous plane superficial to the muscle fascia. Shearing forces disrupt perforating capillaries and lymphatic channels, creating a closed subcutaneous cavity prone to fluid accumulation and secondary infection [8,9]. Over time, pseudocapsule formation can occur, which prevents resorption and often necessitates surgical evacuation [2]. MLLs characteristically occur in the pelvis, trochanteric region, acetabular fracture sites, thigh, and knee, particularly after high-energy trauma [10]. Although the underlying pathophysiology is well characterized, management remains controversial, spanning conservative options such as compression and percutaneous drainage to sclerodesis and open debridement [9,11]. These lesions demonstrate a high susceptibility to microbial colonization, with multiple series reporting a high proportion of cases with positive wound cultures and an associated increase in perioperative infection rates [3,12]. Untreated or recurrent seroma- or hematoma-type MLLs compromise skin viability and increase infection risk, supporting thorough debridement at the time of fracture fixation or as a staged procedure. Current management algorithms incorporate classification-based approaches to guide conservative versus operative treatment, with lesion size, chronicity, and capsule formation serving as critical determinants [2]. Broadly, acute, small, non-capsulated lesions may respond to compression or aspiration, whereas chronic, encapsulated, or infected lesions typically require open evacuation, capsulectomy, and debridement. Within this framework, the present case is classified as an acute, large, non-encapsulated, infected MLL, placing it squarely in the operative management arm requiring open debridement and definitive wound coverage.

Diagnosis of MLL continues to rely heavily on physical examination and eliciting a reliable history to identify a shearing mechanism of injury. In this patient, a clear history was unavailable until the diagnosis became evident. Adjuncts to making the diagnosis included routine and advanced imaging, which proved particularly valuable given the equivocal clinical presentation. Plain radiographs helped exclude osseous injuries, and CT, along with ultrasonography, provided additional diagnostic information. In acute presentations, contrast-enhanced CT may identify active bleeding. In delayed cases, hematomas may coagulate and become more difficult to delineate. Given these limitations, MRI remains the modality of choice, offering superior tissue characterization and precise definition of lesion extent [13]. Lesions typically appear homogeneous and hypointense on T1-weighted imaging and hyperintense on T2-weighted imaging [14]. In this case, an MRI was not obtained due to the acute presentation and emergent need for surgical intervention, reflecting the practical limitations that may preclude its use despite being the diagnostic gold standard. MLL should remain in the upper extremity surgeon’s differential for patients presenting with unexplained swelling post trauma.

Recognition of complications such as skin necrosis and hematoma infection is equally important, as both warrant timely surgical intervention. In this case, intraoperative cultures isolated group A Streptococcus (*S. pyogenes*), a pathogen associated with necrotizing soft-tissue infections and rapid systemic progression if untreated [15]. The precise source of infection was uncertain. The lesion originated from traumatic shearing forces, with subsequent cutaneous necrosis likely providing a microbial entry point for skin flora. Hematogenous seeding was excluded by a negative echocardiogram, although prior IVDU could not be entirely excluded as a contributing factor. This uncertainty does not diminish the surgical principles applied, as management of infected MLLs is dictated by lesion characteristics and timing of injury rather than the infectious source.

This report adds to the limited body of evidence on UE MLLs by illustrating the diagnostic and therapeutic complexities encountered in the forearm. Incomplete history and equivocal imaging contributed to diagnostic ambiguity, initially obscuring the distinction between an infected MLL and a primary abscess. Once infection and necrosis were evident, prompt surgical management was required to achieve source control. Reconstruction with a skin substitute, rather than STSG, was selected in light of patient preference, wound bed compromise, and concerns regarding follow-up adherence, ultimately achieving durable closure despite high-risk factors. Collectively, these insights emphasize the necessity of tailoring management strategies to both lesion characteristics and patient-specific risks, advancing surgical decision-making in rare presentations of MLL.

## Conclusions

UE MLLs are rare and frequently misdiagnosed due to their nonspecific presentation and anatomic atypicality. Delayed history, equivocal imaging, and clinical overlap with cellulitis or abscess can obscure diagnosis and postpone definitive care. Forearm swelling following trauma should prompt consideration of closed degloving injury, particularly when fluctuance, progressive enlargement, or skin compromise is present.

Once infection or necrosis is evident, timely surgical intervention is essential to achieve source control and preserve soft-tissue viability. Management should be individualized based on lesion characteristics and patient-specific risk factors. In this case, use of a dermal allograft provided durable closure in a high-risk patient who declined STSG, demonstrating a viable reconstructive alternative. Early recognition and tailored operative strategy are critical to reducing morbidity in anatomically uncommon presentations such as the forearm.

## References

[1] Cochran GK, Hanna KH (2017). Morel-Lavallee lesion in the upper extremity. Hand (N Y).

[2] Dawre S, Lamba S, Sreekar H, Gupta S, Gupta AK (2012). The Morel-Lavallee lesion: a review and a proposed algorithmic approach. Eur J Plast Surg.

[3] Scolaro JA, Chao T, Zamorano DP (2016). The Morel-Lavallée lesion: diagnosis and management. J Am Acad Orthop Surg.

[4] Vanhegan IS, Dala-Ali B, Verhelst L, Mallucci P, Haddad FS (2012). The Morel-Lavallée lesion as a rare differential diagnosis for recalcitrant bursitis of the knee: case report and literature review. Case Rep Orthop.

[5] Nickerson TP, Zielinski MD, Jenkins DH, Schiller HJ (2014). The Mayo Clinic experience with Morel-Lavallée lesions: establishment of a practice management guideline. J Trauma Acute Care Surg.

[6] Ab Halim MAH, Rampal S, Devaraj NK, Badr IT (2020). A peculiar case of Morel-Lavelle lesion of upper limb. Med J Malaysia.

[7] Patel A, Wee C, Ng MK, Kumar A, Harvey D (2020). Novel use of a closed liposuction system: treatment of an acute Morel-Lavallée lesion of the upper extremity. Cureus.

[8] Sarrami SM, Douglas N, McGraw I, Parent B, Cruz C (2024). Morel-Lavallee associated lymphedema treated with lymphovenous anastomosis: a case report. Injury.

[9] Jones RM, Hart AM (2012). Surgical treatment of a Morel-Lavallée lesion of the distal thigh with the use of lymphatic mapping and fibrin sealant. J Plast Reconstr Aesthet Surg.

[10] Tejwani SG, Cohen SB, Bradley JP (2007). Management of Morel-Lavallee lesion of the knee: twenty-seven cases in the national football league. Am J Sports Med.

[11] Tseng S, Tornetta P 3rd (2006). Percutaneous management of Morel-Lavallee lesions. J Bone Joint Surg Am.

[12] Hak DJ, Olson SA, Matta JM (1997). Diagnosis and management of closed internal degloving injuries associated with pelvic and acetabular fractures: the Morel-Lavallée lesion. J Trauma.

[13] Sulaiman SR, Alsuhaymi AM, Al-Zubaidi SA, Almusallam AA, Yassin AM, AlArabi R (2022). Morel-Lavallée lesion of the elbow region ‎in a young male: case report and ‎literature review. Cureus.

[14] Nair AV, Nazar P, Sekhar R, Ramachandran P, Moorthy S (2014). Morel-Lavallée lesion: a closed degloving injury that requires real attention. Indian J Radiol Imaging.

[15] Green RJ, Dafoe DC, Raffin TA (1996). Necrotizing fasciitis. Chest.

